# Effective Zearalenone Degradation in Model Solutions and Infected Wheat Grain Using a Novel Heterologous Lactonohydrolase Secreted by Recombinant *Penicillium canescens*

**DOI:** 10.3390/toxins12080475

**Published:** 2020-07-25

**Authors:** Larisa Shcherbakova, Alexandra Rozhkova, Dmitrii Osipov, Ivan Zorov, Oleg Mikityuk, Natalia Statsyuk, Olga Sinitsyna, Vitaly Dzhavakhiya, Arkady Sinitsyn

**Affiliations:** 1 All-Russian Research Institute of Phytopathology, Bolshie Vyazemy, 143050 Moscow, Russia; mod-39@list.ru (O.M.); dzhavakhiya@yahoo.com (V.D.); 2 Federal Research Centre “Fundamentals of Biotechnology” of the Russian Academy of Sciences, 119071 Moscow, Russia; a.rojkova@fbras.ru (A.R.); doosipov@gmail.com (D.O.); inzorov@mail.ru (I.Z.); apsinitsyn@gmail.com (A.S.); 3 Chemistry Department, M.V. Lomonosov Moscow State University, 119991 Moscow, Russia; oasinitsyna@gmail.com

**Keywords:** zearalenone, decontamination, enzymatic degradation, lactonohydrolase, enzyme preparations, recombinant proteins, safe agricultural products, improving the feed nutritional value

## Abstract

This paper reports the first results on obtaining an enzyme preparation that might be promising for the simultaneous decontamination of plant feeds contaminated with a polyketide fusariotoxin, zearalenone (ZEN), and enhancing the availability of their nutritional components. A novel ZEN-specific lactonohydrolase (ZHD) was expressed in a *Penicillium canescens* strain PCA-10 that was developed previously as a producer of different hydrolytic enzymes for feed biorefinery. The recombinant ZHD secreted by transformed fungal clones into culture liquid was shown to remove the toxin from model solutions, and was able to decontaminate wheat grain artificially infected with a zearalenone-producing *Fusarium culmorum*. The dynamics of ZEN degradation depending on the temperature and pH of the incubation media was investigated, and the optimal values of these parameters (pH 8.5, 30 °C) for the ZHD-containing enzyme preparation (PR-ZHD) were determined. Under these conditions, the 3 h co-incubation of ZEN and PR-ZHD resulted in a complete removal of the toxin from the model solutions, while the PR-ZHD addition (8 mg/g of dried grain) to flour samples prepared from the infected ZEN-polluted grain (about 16 µg/g) completely decontaminated the samples after an overnight exposure.

## 1. Introduction

For current high-productive agriculture, the provision of the safety of feedstuffs and the improvement of their nutritional value are important and interrelated challenges, which require an integrated approach. Several strategies were developed in recent decades to solve these problems including the enzymatic decontamination of mycotoxin-polluted agricultural feedstock, as well as the removal of anti-nutritional factors from silage, hay, fodder or other feeds, using enzyme preparations [1,2]. However, there is still a need in the development of new remedies with the involvement of innovative advances to promote the production of safe feeds possessing high nutritional value. 

The toxicological safety of plant and livestock products is a prerequisite for food safety. Mycotoxins produced by Fusarium fungi belong to well known feed pollutants, which are able to enter the food chains and induce serious risks for human and animal health [3] as well as result in significant economic losses. One of the most common fusariotoxins, zearalenone (ZEN) [4,5], is synthesized through a polyketide pathway as a secondary metabolite in several common *Fusarium* species (e.g., *F. culmorum*, *F. graminearum*, and also *F. avenaceum*, *F. poae*, *F. equiseti*), which damage grain cereals, forage grasses, legumes, vegetables, and other economically significant crops in regions of temperate or warm climate including Russia [6,7,8]. These fungi keep growing on forage grasses and grain during storage, causing post-harvest contamination with ZEN [9]. This mycotoxin is highly thermostable, insensitive to UV irradiation, is not destroyed during feed or food processing, and remains toxic after all these exposures [10,11]. It can be broken chemically only in strong alkalis or oxidants, the use of which is quite limited because of the obvious drawbacks [12]. In contrast to steroid hormones, ZEN is a nonsteroidal compound (a macrocyclic lactone of β-resorcylic acid [13]), but possesses a high estrogenic activity, disrupts the functioning of reproductive and endocrine systems in humans and animals [10,11], as well as suppresses the immunity [14,15]. 

Since ZEN is a feed and food contaminant of a considerable concern, the development of methods for its effective detoxification, which would provide keeping the quality of the treated feedstuffs and minimally affect the environment, is of a special attention. A large cluster of such investigations is focused on ZEN transformation or degradation to non-estrogenic derivatives [10,13] using microbes [16,17,18,19,20] or isolated enzymes [1,21,22,23,24], especially lactonase (lactonohydrolase) from *Clonostachys rosea* [25,26,27]. The enzyme ability to open the lactone ring in a toxin molecule and to deprive the ZEN of the estrogenic activity is well documented [19,28,29,30]. A number of studies, which describe the ZEN derivatives formed after the enzymatic reaction, as well as a *zhd101* gene cloning and ZHD101 expression in some prokaryotes and eukaryotes [27,28,31,32], suggested that recombinant lactonohydrolases initially originated from *C. rosea* could be used as an efficient remedy for ZEN decontamination. 

A few years ago, we showed that *Clonostachys rosea* (the former name *Gliocladium roseum*) strain GRZ7, produced extracellular metabolites destroying aflatoxin B1 [33], one of polyketide mycotoxins, the molecule of which contains the lactone ring; we also recently detected a ZEN-catabolizing activity of these metabolites [34]. Based on the mentioned data, GRZ7 was selected for the current research as a source zearalenone-specific lactonohydrolase. The lactonase gene transfer into microbial strains used for feed supplementation with exogenous enzymes seems a promising biotechnology allowing a simultaneous improvement of both the nutritional values of feed ingredients and the safety of these products. Therefore, the present work was aimed at obtaining and studying the target activity of a novel enzyme preparation, amended with recombinant GRZ7 lactonase that was produced by *Penicillium canescens* possessing the developed system of the biosynthesis of extracellular enzymes. There is an auxotrophic form of the *P. canescens* strain PCA-10 that can be transformed by plasmid DNA with exogenous genes. We developed PCA-10 as a host strain intended for the production of heterologous enzymes for food, feed, and renewable biomass biorefinery [35] and created a number of novel efficient recombinant strains and enzyme preparations (different cellulases, hemicellulases, pectin lyases, and inulinases) [36,37,38]. Our studies demonstrated that the rates of the culture growth and biosynthesis of extracellular enzymes in *P. canescens* are rather high, the fermentation medium is simple in composition and inexpensive, and the fermentation process can be easily scaled. *P. canescens* secrets only trace amounts of proteases, thus reducing the possibility of a proteolysis of the produced heterologous proteins. All the aforementioned features make the PCA-10 strain economically and technologically favorable for the production of extracellular enzymes [35]. The expression system of *P. canescens* allows the use of three different inducible promoters (xlnA, bgaS and axhA) to develop heterologous and multicopy homologous expression in host *P. canescens* strains [39]. The ability of *P. canescens* to express *P. verrucolosum* cellulases degrading non-starch polysaccharides was demonstrated in [40]. It was shown that the presence of its own xylanase [41] makes the process be the most efficient. Moreover, the *P. canescens* expression system was used to produce homologous pectinlyase [42] and alpha-galactosidase [43], as well as heterologous *Aspergillus* sp. inulinases [44].

## 2. Results

### 2.1. Cloning and Expression of Zhd101 Gene from C. rosea GRZ7 in Heterologous Hosts

**Figure 1:**
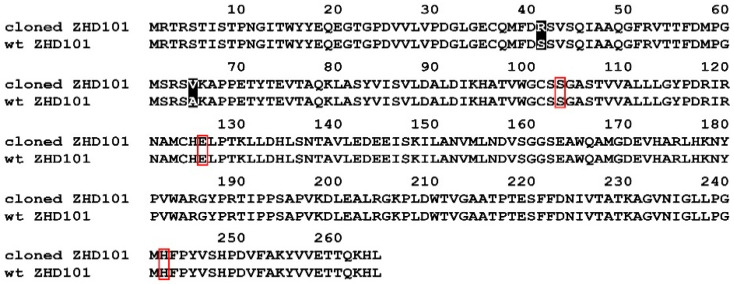
The alignment of the deduced amino acid sequences encoded by the cloned *zhd101* from *C. rosea* GRZ7 and wild-type *zhd101*. Differences due to the substitution of amino acid residues are shaded. The catalytic triad is indicated with a red box.

Cloning of the *zhd101* gene from the strain GRZ7 genomic DNA was based on the known polynucleotide sequence of the zearalenone hydrolase gene from *C. rosea* (GenBank: AB076037.1 [26]). The amplified PCR fragment was 765 bp in length, which corresponds to the size of the *zhd101* gene. For the sequence determination, at least four independent clones were analyzed for each amplified product in order to eliminate any PCR errors. The sequencing of the *zhd101* gene from the strain GRZ7 in both directions revealed a set of mutations in the codons, which did not lead to a change in the amino acid sequence of ZHD101 and was apparently associated with a different codon usage in the tested GRZ7 and other *C. rosea* strains [26]. However, two significant differences were found in the triplets GCC → GTG and AGC → CGC, leading to substitutions in the primary amino acid sequence of S41R and A65V, respectively (Figure 1). No additional substitutions were found in the amino acid sequence ZHD101 from the strain GRZ7.

**Figure 2:**
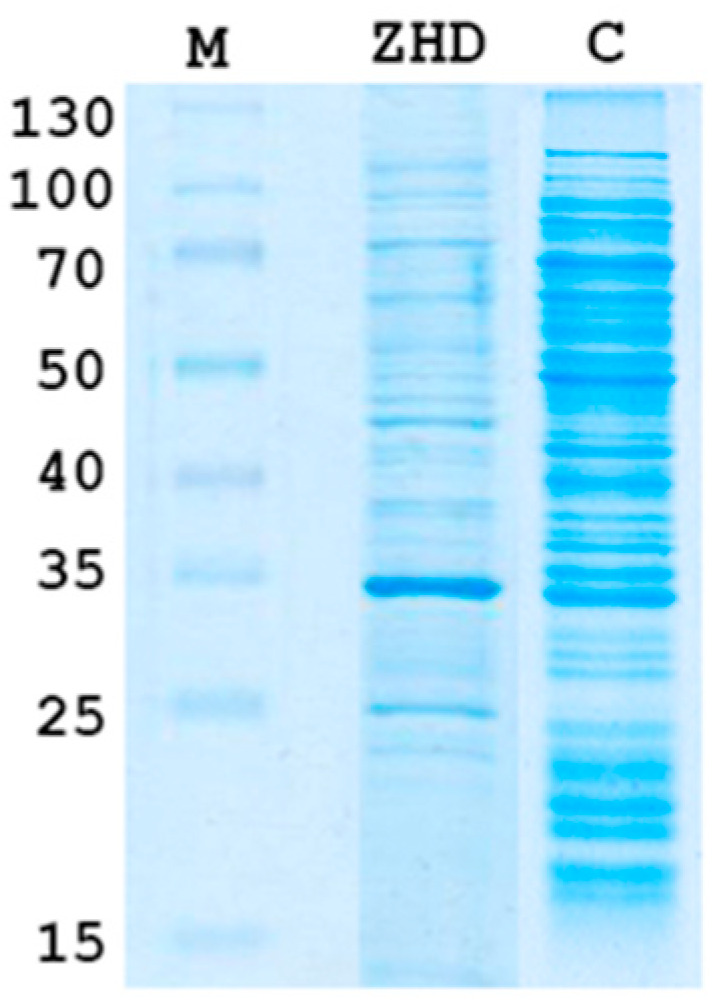
SDS-PAGE of cell lysate after a 0.25 mM isopropyl-β-D-thiogalactoside induction (12% w/v gel). ZHD101, a sample of the *E. coli* RosettaTM (DE3) pLysS strain transformed by the pNIC-ZHD plasmid; C, sample of THE non-transformed *E. coli* RosettaTM (DE3) pLysS strain; M, molecular mass marker proteins (in kDa, ThermoScientific).

The sequence analysis of the *zhd101* gene showed that the coding region was not interrupted by introns. Therefore, to obtain primary information on the *zhd101* gene expression, the expression system of the *E. coli* RosettaTM (DE3) pLysS strain was selected under the control of the T7 promoter. SDS-PAGE lysates after a 0.25 mM isopropyl-β-D-thiogalactoside (IPTG) induction showed the presence of a protein with the mass of 32 kDa (Figure 2). 

**Figure 3:**
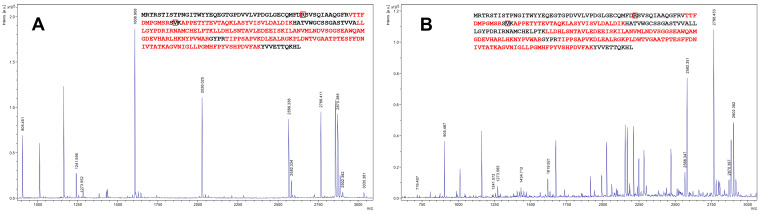
Results of a MALDI-TOF analysis of ZHD101 from the strain GRZ7. The matching four tryptic peptides are marked in bold red, and the substitutions in 41 and 65 positions are squared in red boxes. (**A**) ZHD101 expressed in the *E. coli* RosettaTM (DE3) pLysS strain. (**B**) ZHD101 expressed in the PCA-10 strain.

ZHD101 was identified using the MS peptide fingerprinting technique. The mass spectrum of the ZHD101 is shown in Figure 3A; the matching four tryptic peptides are marked in bold red. The HPLC-analyses of the cell lysates obtained from three randomly selected transformed *E. coli* clones confirmed the target activity of the recombinant ZHD101. No ZEN-corresponding peak was detected in all lysates tested after an overnight incubation at 25–27 °C and pH 8.3, while 99.4 ± 0.21% of a ZEN standard added before the incubation into a similar lysate of non-transformed *E. coli* cells was found.

Considering all the advantages of the fungal expression system described above, it was decided to obtain a recombinant strain producing zearalenone hydrolases based on the highly productive *P. canescens* PCA-10 host strain under the control of an inducible xylanase promoter (*xylF*). For this, the *zhd101* gene from the strain GRZ7 was cloned using the previously developed PC1 [35] and a synthetic gene designed with the codon usage of *P. canescens* PCA-10. Presumably, the use of a synthetic gene would increase the level of heterologous expression [45].

The target protein ZHD101 was successfully expressed and secreted by recombinant *P. canescens*. The SDS-PAGE analyses of the cell-free supernatants of the transformant culture indicated that the molecular weight of the secreted ZHD101 was approximately 30 kDa that corresponds to its mass, theoretically calculated based on its primary sequence (data not shown). The MS analysis also confirmed the presence of the ZHD101 protein in the analyzed supernatants (Figure 3B). As expected, no target protein was found, when the supernatants of the *P. canescens* host strain PCA-10 were preliminary analyzed by these methods.

In both heterologous systems, lactonohydrolase was found to be expressed more effectively than in the host strain, and *P. canescens* ZHD101 showed the highest productivity. The average content of the target enzyme in 1 mL of culture liquid (CL) of the transformed *P. canescens* was 0.11 mg per mg of total protein secreted into CL, while 1 mL of bacterial supernatant contained 0.02 mg of the recombinant ZHD101 per mg of lysed *E. coli* cells; the productivity of *C. rosea* GRZ7 was almost two orders of magnitude less compared to that of *P. canescens* ZHD101.

The freeze-dried supernatants of a culture liquid of the recombinant *P. canescens* (hereinafter referred to as PR-ZHD) were used as preparations for the enzymatic degradation of ZEN in model solutions and wheat grain artificially infected by a ZEN-producing *F. culmorum* strain.

### 2.2. Enzymatic Degradation of Zearalenone in Model Solutions and Wheat Grain Infected with a Toxigenic F. culmorum

The degradation dynamics of ZEN in model buffer solutions containing PR-ZHD (2 mg/mL) of three randomly selected fungal clones were investigated, using co-incubations of the toxin and the recombinant protein, at different pH values and temperatures in time course experiments. The tested clones possessed practically equal efficacy towards the ZEN removal from the model solutions, and this process was significantly influenced by both factors studied.

**Figure 4:**
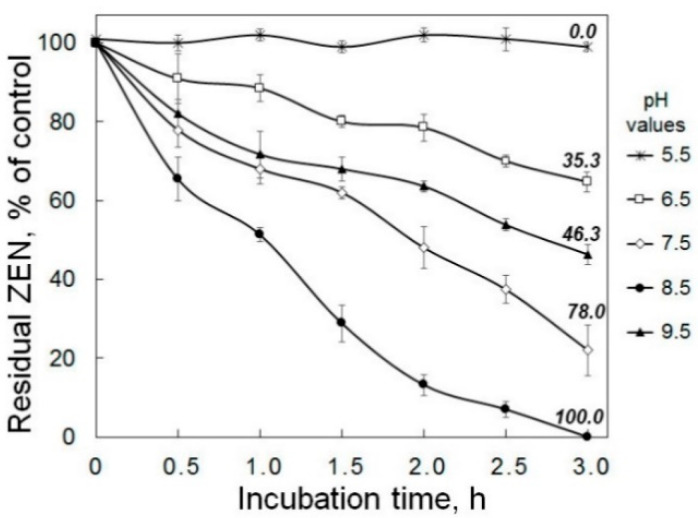
The effect of pH on the dynamics of zearalenone (ZEN) degradation in model solutions by recombinant lactonohydrolase expressed in *Penicillium canescens*. The enzyme preparations tested (PR-ZHD) represented the freeze-dried supernatants of culture liquid (CL) of the transformed *P. canescens* PCA-10, which were dissolved in buffer solutions with various pH values. The numbers above the curves indicate the percentage of ZEN reduction at the end of the co-incubation with PR-ZHD at 30 °C compared to the control (ZEN in the corresponding buffer, see Materials and Methods, 6.3). Results expressed as the mean of three experiments ± SE (*n* = 3). The representative data are out of one of three transformed PCA-10 clones.

**Figure 5:**
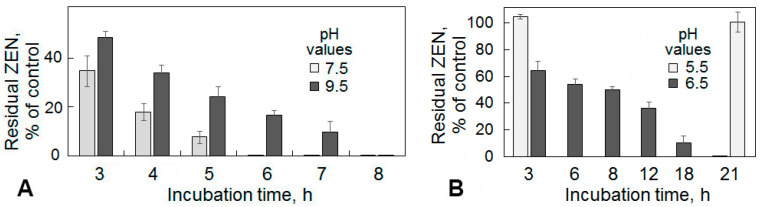
Histograms illustrating the time course of a zearalenone (ZEN) degradation in model solutions by the recombinant lactonohydrolase preparation occurred at non-optimal alkaline (**A**) and acidic pH (**B**) values as well as the irreversible inactivation of the enzyme at pH 5.5 (**B**). See explanations in Figure 4.

In general, PR-ZHD showed the ZEN-degrading activity in the pH range from 6.5 to 9.5, but the most rapid ZEN removal from the solutions was observed when it was co-incubated with PR-ZHD at pH 8.5. In this case, no detectable amount of the toxin was found already after a 3 h exposure. The co-incubation at other alkaline pH values (7.5 or 9.5) decreased the ZEN degradation rate (Figure 4) and resulted in a complete removal of the mycotoxin within six or eight hours, respectively (Figure 5A). Under acidic conditions, recombinant ZHD was still able to hydrolyze ZEN at pH 6.5, while carrying out the reaction at pH 5.5 led to an inactivation of the enzyme (Figure 4). In our experiments, only about 35% of the toxin added to the PR-ZHD solutions prior incubation was catabolized within three hours at pH 6.5, and a complete ZEN degradation occurred seven times slower (Figure 5B) than at pH 8.5 (see Figure 4). At pH 5.5, no mycotoxin removal was observed up to the end of 21 h observations (Figure 5B).

**Figure 6:**
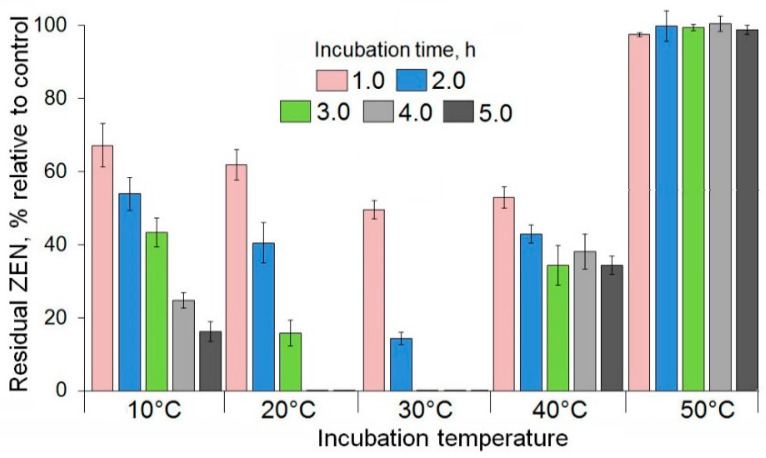
The effect of temperature on the degradation of zearalenone (ZEN) in model solutions after incubation at optimal pH 8.0 with the recombinant lactonohydrolase preparation (PR-ZHD **). Results are presented as the means of three experiments, each including three replications. Error bars indicate SD. ** For additional explanations see captions under Figure 4.

To characterize the effectiveness of a ZEN degradation in the PR-ZHD-containing solutions at different temperatures, the residual toxin was measured each hour during a 5 h incubation at the optimum pH 8.0 (Figure 6). The optimum temperature for the degradation was 30 °C. At this temperature, the ZEN concentration was twice reduced within an hour and reached the non-detectable level after 3 h. The enzyme was also active at room temperature and 10 °C, providing an 84% reduction of the initial ZEN concentration in 3 and 5 h, respectively, as well as a complete degradation at 20 °C within 3 h. A temperature increase resulted in either a rapid reduction of the enzymatic activity (40 °C) or the absolute inactivation of the enzyme (50 °C) (Figure 6). We also found that PR-ZHD remained stable at 4 °C, and its activity was not lost after storage for at least one month (data not shown).

**Figure 7:**
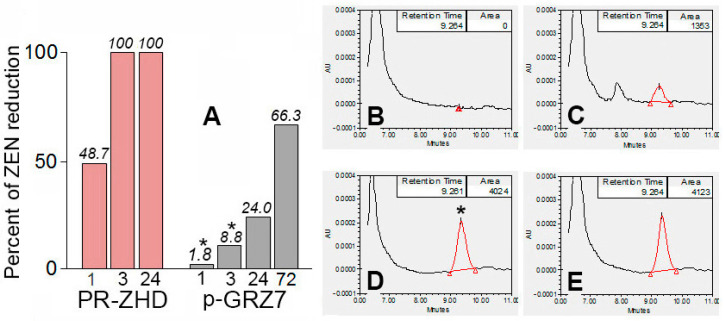
Histogram showing different effectiveness of zearalenone (ZEN) degradation in the preparations of the recombinant lactonohydrolase (PR-ZHD) and ZEN-catabolizing extracellular metabolites obtained from the *C. rosea* strain GRZ7 (p-GRZ7), which was used a source of *zhd101* (**A**); and the corresponding chromatograms illustrating the results of the overnight ZEN incubation in PR-ZHD (**B**), p-GRZ7 (**C**) or a preparation from the non-transformed host *P. canescens* PCA-10 (**D**) obtained similar to PR-ZHD. (**E**) The commercial ZEN standard (Sigma-Aldrich, USA) in 100 mM Tris-HCl buffer after incubation under the same conditions (control to **D**). ZEN was added into the preparations up to the equal concentration (5 µg/mL), while the p-GRZ7 concentration four-fold exceeded that of PR-ZHD. A co-incubation was carried out at pH 8.0 and 30 °C. Asterisks indicate the lack of statistically significant differences between the corresponding controls and treatments at *p* ≤ 0.05.

A comparison of PR-ZHD and a preparation of ZEN-catabolizing extracellular metabolites obtained from the strain GRZ7 (p-GRZ7) used in our research as a source of a zearalenone-specific lactonohydrolase gene showed that the target activity of PR-ZHD was significantly more effective (Figure 7). After a 3 day incubation under optimal conditions, the average reduction of ZEN content exposed to p-GRZ7 used at a concentration four-fold higher than that of PR-ZHD reached 66% (Figure 7A,C). 

To examine if PR-ZHD can serve as a potential tool for the decontamination of a fungal-damaged ZEN-contaminated grain, a pathogenic *F. culmorum* strain able to produce this toxin was grown on autoclaved wheat kernels. The extracts of artificially infected grain were found to contain ZEN. In contrast, these extracts were ZEN-free if the samples of the flour prepared from contaminated grain were co-incubated overnight with PR-ZHD at pH 8.0 and 30 °C (Table 1). No reduction of the toxin content was observed in the extracts from infected kernels, if the flour suspension was exposed to a freeze-dried supernatant from the non-transformed host PCA-10 (pPCA10) under the same conditions. In addition, co-incubation with pPCA10 did not result in any removal of the ZEN standard added to the flour suspension prior to the treatment with this preparation (Table 1).

## 3. Discussion

As with other mycotoxins, numerous physical and chemical methods intended to remove a ZEN fusariotoxin from agricultural products are being developed today. However, only a few of these methods are safe and may be implemented in practice [46]. Biological decontamination based on the use of mycotoxin-degrading microorganisms as feed additives has less limitations, but can be associated with some disadvantages, such as the production of undesirable metabolites and the worsening of the quality of products. However, mycotoxin destroying or biotransformation by extracellular enzyme preparations of microbial origin allows avoiding the drawbacks of a decontamination by living microorganisms. An additional advantage of such preparations is that they can be manufactured by large-scale fermentation and can include not only toxin-degrading enzymes but also other ones possessing alternative useful properties. In this regard, this study focused on the introduction of the ZEN-degrading activity into *P. canescens* PCA-10 producing a complex of endogenous hemicellulases such as xylanases, arabinofuranosidases, β-galactosidase, etc.

We were able to receive several transformed clones expressing and secreting a novel recombinant ZHD101 protein that rapidly removed ZEN from the model solutions and was able to decontaminate the wheat grain infected with a toxin-producing fungus.

According to our experimental data, the ZHD101 amino acid sequence of strain GRZ7 (265 aa) is slightly different from the closest homologue of ZHD101 from *C. rosea*. The amino acid sequence of ZHD101 from GRZ7 showed a high identity (99%) with ZHD101 from *C. rosea* with the putative catalytic triplet SHE motif (Ser102-His242-Glu126) [47]. It should be noted that the Ala65Val substitution found in the new ZHD101 is located in an unstructured region of the protein globule, and the presence of valine at the position 65 can lead to the stabilization of the protein globule due to the hydrophobization of the chain. As for the Ser41Arg substitution, it is located in the α-helix, which can lead to some destabilization; however, this residue is far from the Ser102-His242-Glu126 triad, which determines the catalytic activity of ZHD101 [47] (Figure 1).

In general, our results on determining the pH and temperature dependence of the ZEN-degrading activity of the enzyme secreted by the transformed *P. canescens* PCA-10 coincided with the data reported for the ZHD of *C. rosea* IFO 7063 [26] as well as for the recombinant ZHD101 and ZLHY6, which were expressed in *Escherichia coli* and *Pichia pastoris*, respectively [28,48]. However, ZHD101 expressed in PCA-10 clones had the highest activity at pH 8.5 (similar to ZLHY6) and was much less active under more alkaline conditions, differing from the enzyme of *C. rosea*, which had the maximum activity at pH 9–10 [26]. Moreover, the ZEN removal at pH 7.5 occurred more rapidly than at pH 9.5. Like the aforementioned preparations of lactonohydrolases, PR-ZHD was inactivated at 50 °C and an acidic pH below 6.0. It is important to note that even at 10 °C PR-ZHD was able to degrade 80–86% of the toxin within 5 h of incubation (Figure 6), since this discovery can be significant for a post-harvest decontamination of stored feeds.

The host strain GRZ7 produced much less lactonohydrolase compared to the transformed *P. canescens* PCA-10 clones. Our preliminary experiments [34] revealed the necessity of at least a ten-fold concentration of GRZ7 extracellular metabolites in order to provide a significant level of ZEN degradation. This suggested the productivity of GRZ7 turned out to be insufficient to consider it as a prospective producer of a ZEN-degrading enzyme. At the same time, we showed that *P. canescens* PCA-10 possessed the well developed system for the biosynthesis of extracellular enzymes, as well as some other advantages as a super-producer of various enzyme preparations [36,37,38,39,40,41,42,43,44]. By transforming this active producer of extracellular metabolites, we succeeded in obtaining recombinant *P. canescens*, of which the enzyme preparation showed ZEN-degrading efficacy, significantly exceeding that of the host *C. rosea* strain.

As known, considerable amounts of nutrients and fluids (e.g., xylans and other polysaccharides) are excreted from the animal organisms without being metabolized [49]. It is worth noting that our recent experiments suggest that the freeze-dried supernatants of the transformed PCA-10 retained the ability to destroy polysaccharides. However, further investigations are necessary to confirm the PR-ZHD promise for the application on ZEN-polluted forage wheat with the purpose of simultaneous decontamination and improving the availability of feed nutritional components. Another possible way to provide a highly effective biological protection of agricultural feedstock from contamination is a combination of mycotoxin-degrading enzyme preparations and the natural inhibitors of the toxigenesis [50]. Such preparations might be used for providing an additional feed decontamination in those cases when an inhibitor incompletely suppresses the biosynthesis of mycotoxins. Our studies of PR-ZHD, including the dose-rate effect and testing the enzyme preparation on cereal samples naturally contaminated with ZEN, as well as the assessment of the possibility to combine PR-ZHD with some inhibitors of ZEN and other polyketide mycotoxin biosynthesis, are in progress. 

It should be also noted that the use of the heterologous production system allowed us to simplify obtaining the enzyme, since no additional purification or culture concentration were needed. In addition, the use of enzymatic preparations for mycotoxin degradation is more preferable than the use of toxin-degrading microorganisms, since microorganisms may also produce some undesirable components or consume the substrate impairing its nutritional value. Thus, the results of the current research open avenues for a potential application of recombinant lactonohydrolase in feed production. If large-scale tests on different feed substrates will show good results, and toxicological studies will confirm the safety of the preparation, it can be industrially produced to develop additives increasing the safety of grain and other feeds.

## 4. Materials and Methods

### 4.1. Microbial Strains and Cultivation Media

The *Escherichia coli* MachI^TM^ T1R strain (Thermo Fisher Scientific Inc., Waltman, MA, USA) was used to obtain competent cells in the subcloning experiments. The *Escherichia coli* Rosetta^TM^ (DE3) pLysS strain (Thermo Fisher Scientific Inc., Waltman, MA, USA) was used to obtain competent cells for zhd101 gene expression. The *Penicillium canescens* PCA-10 strain [35] was used as an auxotrophic host strain (Δ*niaD*) in fungal transformation. The *C. rosea* strain GRZ7 producing ZEN-destroying enzymes and earlier isolated from the fungal consortium of toxigenic *Aspergillus flavus* [51], was applied for the isolation of genomic DNA.

Total genomic DNA was isolated from the *C. rosea* GRZ7 mycelium after a 5 day incubation on minimal medium (g/L): MgSO_4_·7H_2_O—0.2, NH_4_NO_3_—1.0, K_2_HPO_4_—0.9, KCl—0.15, FeCl_2_—0.002, ZnSO_4_—0.002, glucose—5.

The screening of transformants was carried out in shake flasks (total volume 500 mL, fermentation broth volume 100 mL). *E. coli* recombinant strains were cultivated using standard Luria–Bertani broth supplemented with 50 µg/mL kanamycin, 34 µg/mL chloramphenicol and 0.25 mM isopropyl β-D-thiogalactoside for expression induction.

*P. canescens* cultivation media contained (g/L): soybean hulls—45, corn extract—50, KH_2_PO_4_—15.

### 4.2. Expression of Zhd101 Gene in Heterologous Hosts and ZHD101 Identification

The coding region of the *zhd101* gene (GenBank AN: AB076037.1) was amplified by PCR with the ZHD-LIC5 and ZHD-LIC3 primer pair (Table 2) and the GRZ7 genomic DNA isolated using a QIAquick Plant Mini Kit (QIAGEN, Valencia, CA, USA) as a template. The obtained PCR product (765 bp) was cloned into pNIC-Bsa4 vector (a gift from Opher Gileadi, Addgene plasmid #26103 [52]) using the ligation independent cloning protocol [53]. The resulting pNIC-ZHD plasmid (Figure S1A, see Supplementary Materials) was isolated using a QIAquick Miniprep Kit (QIAGEN, Valencia, CA, USA). The desirable *zhd101* insertion in a pNIC-Bsa4 vector was confirmed by the DNA sequencing of a full-size pNIC-ZHD plasmid. Then pNIC-ZHD was used to produce the recombinant ZHD101 in *E. coli* Rosetta^TM^ (DE3) pLysS competent cells.

The screening of *E. coli* transformants was carried out in the Luria–Bertani (LB) broth 12 h after induction with 0.25 mM IPTG. The ZHD101 expression was characterized by SDS-PAGE in 12% gel using a Mini Protean II equipment (Bio-Rad Laboratories, USA) and a PageRuler Unstained Broad Range Protein ladder (ThermoFisher Scientific, Waltham, MA, USA). For the enzyme identification, pieces of protein bands were cut off the electrophoretic gel, digested with trypsin according to a standard protocol [54], and the resulting peptide mixture was analyzed by MALDI-TOF mass spectrometry (MS) using an UltrafleXtreme mass spectrometer (Bruker Daltonik GmbH, Bremen, Germany). The MS data were analyzed using the online MASCOT service (http://www.matrixscience.com/).

A codon analysis of the *zhd101* gene from GRZ7 was carried out using a Sequence Manipulation Suite (www.bioinformatics.org/sms2/codon_usage.html). A synthetic *zhd101* gene was designed using a GenScript program (www.genscript.com/gensmart-free-gene-codon-optimization.html) based on the *Aspergillus niger* codon usage.

The cloning of the synthetic *zhd101* gene (Evrogen, Moscow, Russia) into the *P. canescens* PCA-10 (Δ*niaD*) auxotrophic strain was carried out as described previously [55,56]. The pair of oligonucleotides, ZHD-UpLIC and ZHD-LowLIC, was constructed to amplify the *zhd101s* gene. Briefly, the *zhd101s* gene was cloned into a modified linear PC1 shuttle vector containing a promoter and the terminator regions of the *xylA* gene-encoding xylanase A from *P. canescens* [35]. Then, the resulting expression plasmid pXEG-ZHD (Figure S1B) was transformed into competent *E. coli* MachI cells, and the produced DNA material was supplied for analysis. The *E. coli* MachI cells were cultured as described elsewhere [56]. The absence of additional mutations, deletions, or insertions in the *zhd101s* gene was confirmed by its sequencing in both directions according to Sanger et al. [57].

The pXEG-ZHD plasmid was directed into the protoplasts of the host *P. canescens* PCA-10 (Δ*niaD*) strain together with a pSTA10 plasmid (10:1, µg) using the modified method described by Aleksenko et al. [58]. The efficiency of integration amounted to 15–20 clones per 1 µg of the target DNA that corresponded to a standard frequency of the transformation for the fungal genus *Penicillium* [58]. Stable transformants were grown in shake flasks for 6 days using a small volume (100 mL) of the liquid cultivation medium as described above. 

The screening of recombinant *P. canescens* ZHD strains was carried out directly from colonies with the use of a routine PCR protocol and Phire Hot Start II DNA Polymerase (ThermoFisher Scientific, Waltham, MA, USA) (Figure S2).

The selected clones were cultivated in a 3 L glass KF-104/3 fermenter (Prointex, Russia). The medium composition for *P. canescens* fermentation was identical to that in shake flasks (see above). Fermentation was carried out for 144 h. Finally, the culture broth was centrifuged at 4000 rpm for 20 min on an Avanti JXN-26 centrifuge (Beckman Coulter, Brea, CA, USA) to remove the biomass and insoluble components of the nutrient medium. The supernatant was freeze-dried on a VirTis BenchTop 2K ES freeze dryer (SP Scientific, Warminster, PA, USA). The resulting lyophilizate was referred to as the ZHD recombinant preparation (PR-ZHD). Additionally, a freeze-dried supernatant of the culture liquid prepared under similar fermentation conditions was obtained from a non-transformed recipient PCA-10 strain.

### 4.3. Estimation of the Zearalenone Removal from Model Solutions under Different Conditions

PR-ZHD samples were dissolved in 100 mM Tris-HCl buffer with a pH ranging from 6.5 to 9.5, or 100 mM acetate buffer with pH 5.5 to a final concentration of 2 mg/mL, and sterilized by passing through a 0.22 µm Millipore filter. The sterilized PR-ZHD solutions were supplemented with a commercial ZEN (Sigma-Aldrich, St. Louis, MO, USA) dissolved in a minimum volume of methanol up to a final concentration of 5.0 µg/mL and incubated at 30 °C for different time intervals until a complete ZEN removal from the incubation solution. Following that, the effect of different temperatures (10–50 °C for 5 h) on the degradation process at the optimal pH was investigated. The reaction was terminated by 1N HCl. The corresponding preparation obtained from non-transformed PCA-10 (pPCA10) was used as the control. A freeze-dried sample of a ZEN-degrading fraction of GRZ7 extracellular metabolites (p-GRZ7) with a final concentration of 8 mg/mL, which was prepared by ultrafiltration, precipitation with ammonium sulfate, and partial purification by the anion-exchange chromatography [26,33], was used to compare the ZEN degradation by the recombinant ZHD and the metabolites of the initial *C. rosea* strain.

To confirm the ZEN-degrading activity of ZHD produced in the *E. coli* expression system, the fresh lysates of the transformed *E. coli* clones were examined after an overnight incubation at 27–28 °C and pH 8.3 [59].

### 4.4. Decontamination Test

To contaminate the wheat grain with ZEN, 2.0 mL of a macroconidial suspension (10^6^ conidia/mL) of the toxin-producing *F. culmorum* strain grown on Chapek agar was added into 250 mL flasks (in three replications) each containing 50 g of moistened autoclaved wheat grain. The flasks were incubated at 25–26 °C for 14 days. The infected grain was dried at 40 °C to a constant weight and powdered in a laboratory mill. Randomized flour samples (5 g) from each flask were placed into 250 mL flasks, suspended in 60 mL of a 100 mM Tris-HCL buffer (pH 8.5) and supplemented with PR-ZHD at a rate of 8 mg per gram of dried grain. After an overnight incubation at 30 °C at a slight automated agitation, acetonitrile was added to a final concentration of 84%, and the suspensions were extracted at vigorous shaking for 30 min. ZEN was further purified from the extract as described earlier [60]. The precipitate obtained after the evaporation of the purified ZEN-containing extract on a rotary evaporator was dissolved in a minimum volume of a methanol–acetonitrile mixture (1:1) and analyzed by reversed-phase HPLC.

### 4.5. Quantification ZEN Residues

The residual ZEN content in the model solutions or the extracts from ZEN-contaminated wheat was analyzed using a Waters1525 Breeze HPLC system equipped with a Waters 2487 UV detector (Waters Corp, USA). Aliquots of the tested solutions (10 µL) were applied on a Symmetry C18 (5 µm, 150 × 4.6 mm) temperature-controlled (27 °C) column (Waters Corp., Milford, MA, USA), eluted with a acetonitrile–methanol–water mix (1:1:0.75) at a rate of 0.8 mL/min, and detected at 254 nm. To determine the percent of recovery, the ZEN standard (Sigma-Aldrich, St. Louis, MO, USA) was added into a sample of non-infected wheat grain (4 µg per 1 g of the milled grain) prior to the extraction. The limit of detection in the wheat grain extracts was 0.2 mg/kg, while the recovery amounted to 79%. The linear dependence of the peak area on the analyte amount was observed at a sensitivity of 0.01 units of the absorption/scale.

### 4.6. Statistical Analysis

Tests on the ZEN degradation in the model solutions or infected grain samples included three replications per treatment. Quantitative results were analyzed by a STATISTICA v. 6.1 software (StatSoft Inc., Tulsa, OK, USA) and presented as the mean values of the measurements obtained in at least three independent experimental series. The significance of differences (*p* < 0.05) of the means between treatments and controls were determined using a *t*-test for the independent variables. 

## Figures and Tables

**Table 1:** Zearalenone (ZEN) content in the extracts of autoclaved wheat grain artificially infected with ZEN-producing *F. culmorum*.

Treatments	ZEN, µg/g (M ± SE)
Infected grain (control)	15.58 ± 3,07
Infected grain + PR-ZHD*	<0.2 **
Infected grain + pPCA10	15.73 ± 4.35
Non-infected grain + ZEN ***	3.15 ± 0.09 ***
Non-infected grain + ZEN + pPCA10	3.17 ± 0.13

* PR-ZHD was added to a final concentration of 8 mg/g of dried grain. ** The limit of ZEN detection in the grain extracts was 0.2 µg/g. *** Prior to treatment with pPCA10, the samples were supplemented with an aliquot of a ZEN standard (Sigma-Aldrich, St. Louis, MO, USA) to a final concentration of 4 µg/g. The average ZEN recovery was 79%.

**Table 2:** Primers and oligonucleotides used in the study.

Primer Name	Sequence
ZHD-LIC5	5′- TACTTCCAATCCATGCGCACTCGCAGCACAATCTCGAC -3′
ZHD-LIC3	5′- TATCCACCTTTACTGTCAAAGATGCTTCTGCGTAGTTTC -3′
ZHD-UpLIC	5′- CAAACAGAAGCAACCGACACAATGCGCACTCGCAGCACAATCTCGA -3′
ZHD-LowLIC	5′- AGAGCAAGCCGAGCAGGTTCAAAGATGCTTCTGCGTAG -3′

## Supplementary Materials

The following are available online at https://www.mdpi.com/2072-6651/12/8/475/s1, Figure S1: The schematic diagram of the two plasmids for expression into: *E. coli* RosettaTM (DE3) pLysS strain and *Penicillium canescens* PCA-10, Figure S2: Screening of the recombinant *P. canescens* ZHD strains (1–18).
