# Supplementary material for: Effective Zearalenone Degradation in Model Solutions and Infected Wheat Grain Using a Novel Heterologous Lactonohydrolase Secreted by Recombinant Penicillium canescens
Source: Toxins (Basel). 2020 Jul 25;12(8):475. doi: 10.3390/toxins12080475 (PMC7472149; doi:10.3390/toxins12080475)
Supplement: Supplementary file 1 [file toxins-12-00475-s001.pdf]

Supplementary Materials: Effective Zearalenone Degradation in Model Solutions and Infected Wheat Grain Using a Novel Heterologous Lactonohydrolase Secreted by Recombinant *Penicillium canescens*

Larisa Shcherbakova, Alexandra Rozhkova, Dmitrii Osipov, Ivan Zorov, Oleg Mikityuk, Natalia Statsyuk, Olga Sinitsyna, Vitaly Dzhavakhiya and Arkady Sinitsyn

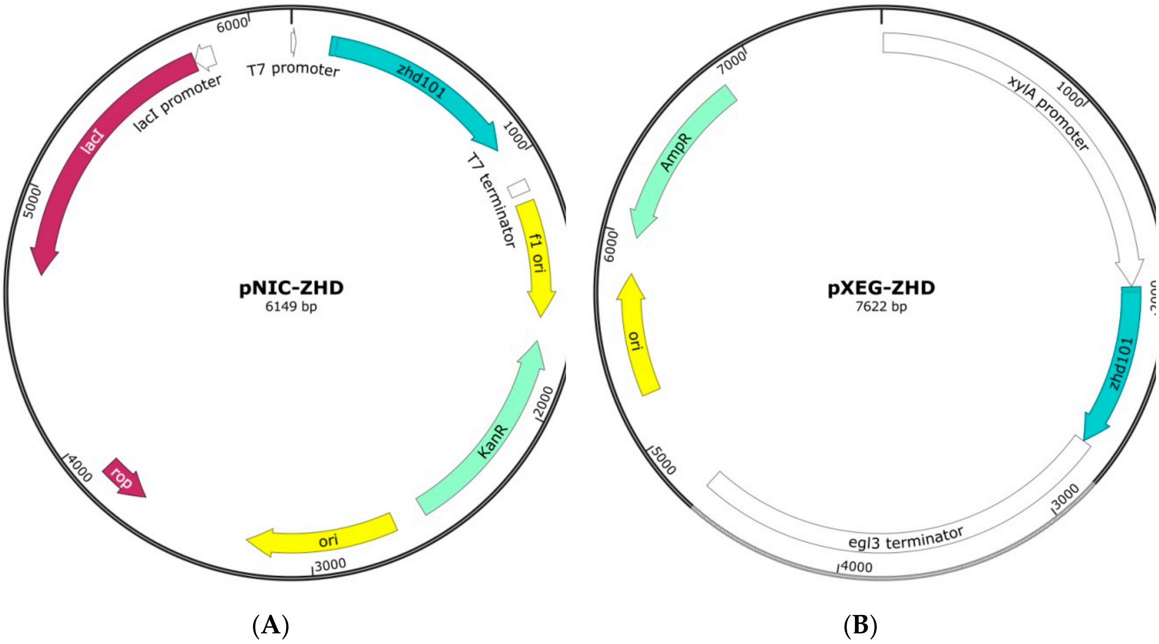

Figure 1. The schematic diagram of the two plasmids for expression into: *E. coli* RosettaTM (DE3) pLysS strain (**A**) and *Penicillium canescens* PCA-10 (**B**).

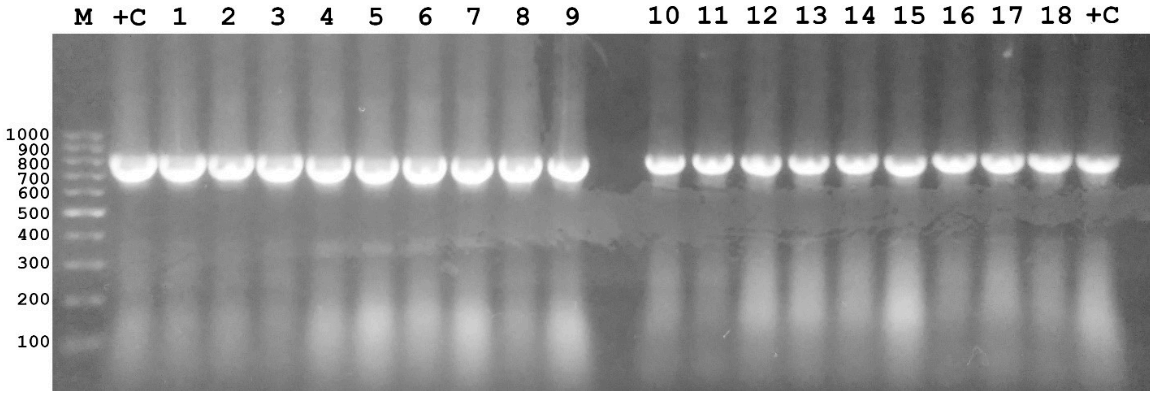

Figure S2. Screening of recombinant *P. canescens* ZHD strains (1–18). Screening was carried out from colonies with using Phire Hot Start II DNA Polymerase (ThermoFisher Scientific, Waltham, MA, USA). +C—positive control from plasmid pXEG-ZHD, M—DNA Ladder (ThermoFisher Scientific, Waltham, MA, USA).
